# Real-world waste dispersion modelling for benthic integrated multi-trophic aquaculture

**DOI:** 10.1371/journal.pone.0303538

**Published:** 2024-05-23

**Authors:** Karl Cutajar, Lynne Falconer, Angus Sharman, Trevor C. Telfer

**Affiliations:** 1 Institute of Aquaculture, University of Stirling, Stirling, Scotland; 2 MFF Ltd., Marsaxlokk, Malta; Central Marine Fisheries Research Institute, INDIA

## Abstract

In real-world situations, marine fish farms accommodate multiple fish species and cohorts within the farm, leading to diverse farm layouts influenced by cage dimensions, configurations, and intricate arrangements. These cage management practices are essential to meet production demands, however, farm-level complexities can impact model predictions of waste deposition and benthic impact near fish cages. This is of particular importance when the cages are used for integrated multi-trophic aquaculture (IMTA) with benthic feeders, where this waste not only affects environmental conditions but also provides a potential food source. The Cage Aquaculture Particulate Output and Transport (CAPOT) model incorporated multiple species, cohorts, and cage arrangements to estimate waste distribution from a commercial fish farm in the Mediterranean between October 2018 and July 2019. This spreadsheet model estimated dispersion for individual fish cages using a grid resolution of 5 m x 5 m. The study categorized discrete production periods for each fish cage every month, aligning with intermittent changes in biomass and food inputs due to different cage management practices throughout production. This approach facilitated the use of detailed input data and enhanced model representativeness by considering variations in cage biomass, food types, settling velocities, and configurations. Model outputs, represented in contour plots, indicated higher deposition directly below fish cages that varied monthly throughout fish production cycles. Deposition footprints reflected changes in cage biomass, food inputs, and farm-level practices reflecting this real-world scenario where aquaculture does not follow a production continuum. Moreover, cohort dynamics and cage movements associated with the cage management practices of the fish farm influenced the quantity and fate of wastes distributed around fish cages, revealing variability in deposition footprints. Clearly, these findings have important implications for the design of benthic IMTA systems, with species such as sea cucumber and polychaetes. Variability in waste deposition creates challenges in identifying where the benthic organisms should be placed to allow optimal uptake of waste to meet their food requirements and increase survivability. Evidently, models have an important role to play and this study emphasizes the need for representative input data to describe actual food inputs, cage biomass changes, and management practices for more representative farm-scale modelling and essentially to improve particulate waste management. To effectively mitigate benthic impacts through IMTA, models must quantify and resolve particulate waste distribution and impact around fish farms to maintain a balanced system with net removal of wastes. Resolving farm-level complexities provides vital information about the variability of food availability and quality for extractive organisms that helps improve recycling of organic wastes in integrated systems, demanding a more representative modelling approach.

## Introduction

Marine fish farms vary considerably in terms of size, husbandry techniques and management practices. Farm layouts can be highly variable between species, production intensity, and location. Different sizes and shapes of cages are used, and even within individual farms there can often be complex or irregularly organised cage systems [1, 2]. The organisation of cages within a farm is one of the major factors that influences impact of waste on the surrounding environment, and a better understanding of different farm layouts could help reduce environmental impact [3]. Further layers of complexity arise in many countries, where multiple fish species are farmed at the same site with minimal organisation of species and size classes within the farm [1, 2, 4]. When multiple cohorts of different species and sizes of fish are stocked at different times in adjacent cages on the same fish farm, a range of cage management practices (e.g. cage batch inputs, cage splitting, cage movement and re-organisation) are required to accommodate production demands. These management practices influence the standing biomass of farmed fish in fish cages and the feeding requirements at the fish farm. Due to these complexities, production is not constant and consequently particulate waste dispersion and deposition near fish cages varies. These practices can present new challenges for predicting waste deposition around these fish farms and implications for management and mitigation of benthic impacts. This is of particular importance when considering the site for coastal integrated multitrophic aquaculture (IMTA), where the waste from fish production is utilised as a nutrient source by lower trophic organisms cultured in the proximity.

Environmental models are used by the aquaculture industry and regulators to help ensure compliance with environmental regulations and evaluate production levels within the ecological carrying capacity of the system [5, 6]. Particle dispersion models simulate the fate and transport of particulate wastes from marine fish cages [7, 8] and predict the benthic impacts of farmed species, including Atlantic salmon (*Salmo salar*) [7, 9, 10], and seabream (*Sparus aurata*) and seabass (*Dicentrarchus labrax*) [4, 11]. These waste dispersion models are often used as decision-support tools that inform aquaculture planning and licensing processes, providing insight into how a farm might impact the environment and what production level may be acceptable within regulatory limits [12]. Models have been used to predict waste deposition from marine fish farms for many years [13], and there have been many advances since the first applications. However, these models have limitations when fish farm management practices, like cage movement or multiple fish species, are not represented in detail [14].

Waste dispersion models tend to use whole farm summaries of feed input, and short-term and averaged data to predict benthic flux from the production of multiple fish species and sizes, within the same farm [4, 7, 10, 15]. Summarised husbandry information can limit model data inputs, and while still valid for simplified production scenarios, detailed input data helps to improve the representativeness of established farm-scale models [4, 16]. Similarly, the use of species-specific information over single averaged data inputs provides better representation of simulated waste deposition from multiple species and cohort fish farms [1, 4]. Where fish farm production is not constant, discrete changes influence the deposition footprint [17, 18]. In some IMTA systems the deposited waste material is consumed by benthic feeders so the modelled footprint would be an indication of food availability and environmental conditions. Hence, simplified scenarios of cohort dynamics and changes in positions of the cage are not enough or appropriate for accurate representation of fish farm deposition footprints needed when used in IMTA with bottom-dwelling extractive species.

Knowledge gaps exist in understanding the variability in fish farm deposition footprints that is associated with the complexities of real-world cage management practices, which has particular importance in setting up deposit-feeding lower trophic species within an IMTA system. The farm-scale spreadsheet-based Cage Aquaculture Particulate Output and Transport (CAPOT) model provides the flexibility to account for complex cage configurations and management practices [19]. In this study, the model was used to predict waste deposition from multiple species from different cohorts farmed at a nearshore fish farm at the centre of the Mediterranean. The distribution of sediment carbon was predicted for discrete periods of production every month established to account for cage management operations carried out at individual cages on the fish farm during production. In our predictions of waste deposition, simulations were based on real-time hydrodynamic conditions, species-specific literature data, discrete food input and cage biomass information, and accounts of cage movements. In the present study, emphasizing the variability in waste deposition around complex fish farming practices contributes towards better predictions of deposition footprints. The work has important implications for effective management and mitigation of benthic impact, particularly in placement and management of deposit-feeding IMTA systems, such as sea cucumbers.

## Materials and methods

### Study site

**Fig 1:**
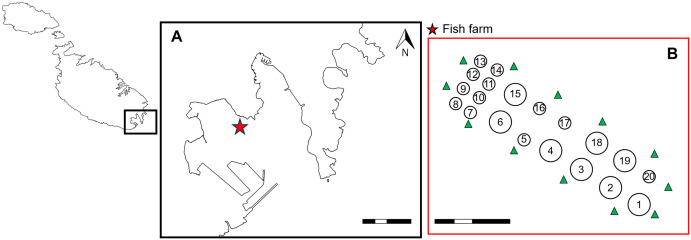
A. Map of Marsaxlokk Bay, the study site located in the southeast of Malta (Scale bar: 1 km). B. Arrangement of fish cages at the fish farm during a specific period of production. Deployment positions of the current profiler around the fish farm indicated by green markers. Grey circles represent fish cages of different dimensions and numbered for position reference. These positions are not always occupied (Scale bar: 100 m).

The study was set up at a nearshore commercial fish farm (35°49’39.90" N, 14°32’30.73" E) in Marsaxlokk Bay, Malta, at the centre of the Mediterranean (Fig 1A). The fish farm is a nursery and juvenile facility for grow-out production. This facility has been in operation since the early 1990s and is run by MFF Ltd. The bay is partly sheltered by a breakwater at the mouth of the bay and is approximately 3.78 km^2^. At the centre of the bay, the nearshore fish farm lies 130 m northwest of navigation channels close to transhipment terminals in this archetypal port area.

At the time of the study, the fish farm reported a total annual production of 719 t and a feed conversion ratio of 1.7. During the study period, commercially available formulated feeds were used for the continual production of sea bream and sea bass juveniles. The juveniles had been transferred as hatchery-produced fingerlings (about 2–3 g) for grow-out at this nearshore aquaculture facility (approximately 13 months at the time of study), before being transferred at about 190 g to an offshore site in deeper waters where they are cultured for approximately 17 months until harvest (harvest size of 550 g). These juveniles are transferred from this shallow and sheltered site to an offshore site in deeper waters where they are cultured until harvest. Moreover, this nearshore fish farm produced small quantities of greater amberjack (*Seriola dumerili*) in one of the fish cages using chopped baitfish fed 2–3 times a week throughout the study period.

The study site has 20 round fish cages that are 12 m or 28 m in diameter. The fish cages have net depths that are between 7 m and 10 m. Fig 1B shows the cage dimensions and the irregular arrangement of fish cages. Water depth was taken from *in-situ* measurements near each fish cage at the fish farm. The fish farm lies on an increasing downward slope into deeper waters in a south-west direction, so that cages 1 to 6 are in 12–13 m water depth, cages 7 to 12 are in 10–11 m, and cages 13 to 20 in 8–9 m.

### Waste dispersion

The dispersion of particulate wastes around the nearshore fish farm in Marsaxlokk Bay was modelled using the highly flexible CAPOT depositional model [19], and compares favourably with established models that are used for environmental regulation (e.g. [7].

**Fig 2:**
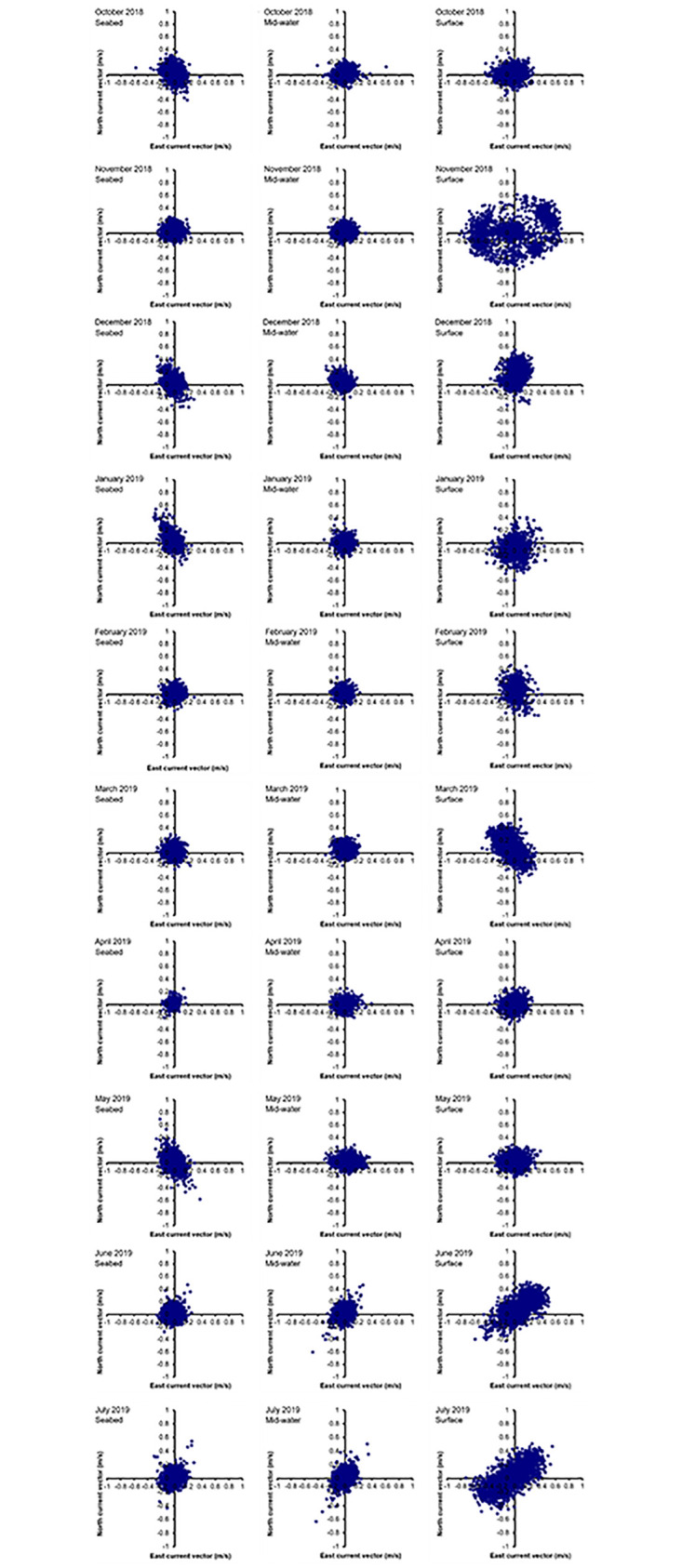
Plots for currents measured near surface, mid-water and near seabed at different positions around the fish farm throughout the study period between October 2018 and July 2019. The water depths at different months were: 7 m in Oct 2018, 13 m in November 2018, 9 m in December 2018, 8 m in January 2019, 12 m in February 2019, 10 m in March 2019, and 9 m in April and May 2019, and 11 m June and July 2019.

The model uses information; hydrographic data, food input and fish biomass data, depth of nets, water depth, and size and arrangement of cages. *In-situ* current speed (m/s) and direction (° N) taken using Acoustic Doppler Current Profiler (Aquadopp Profiler, 400Hz; Nortek, Norway) placed on the seabed, from three bin depths in the water column represented near surface, mid-water, near seafloor currents. Hydrographic data were recorded continuously at 20 min intervals between ten locations around the farm (Fig 1B), within 20 m from the nearest fish cage, over a 10-month period between October 2018 to July 2019 inclusive. Data was extracted every month from the positions around the fish cages. Water currents were predominantly in an east to north direction through the fish farm most of the time and variable, particularly at different depths (as presented in Fig 2). Plots show currents for the study period at the near-seafloor depth (3 m from the seabed), at the near-surface depth (between 7 and 13 m above the seabed) and for mid-water depths (between 4 and 6 m above the seabed). *In-situ measurements* of local currents near fish cages recorded between April 2018 and August 2019 are available through the EMODNET repository at: https://www.emodnet-ingestion.eu/submissions/submissions_details.php?menu=39&tpd=550&step_more=9.

In consideration of the variability in local hydrographic conditions at the site [20], simulations of waste distribution were based on detailed whole-month current datasets at 20 min resolution. This hydrographic data corresponded with the fish production data that was modelled every month to represent the path of initial waste settlement. Wind and tidal influences, and anthropogenic effects, on water movement within the bay have been described in detail in [20].

Within the waste dispersion model, mass balance equations were used to determine the amount of organic carbon and the form of waste dispersed from the fish farm to the surrounding environment [19]. Actual feed input and cage biomass data for sea bream, sea bass and amberjack culture were used within the model to estimate dispersion from fish cages for these farmed species.

**Fig 3:**
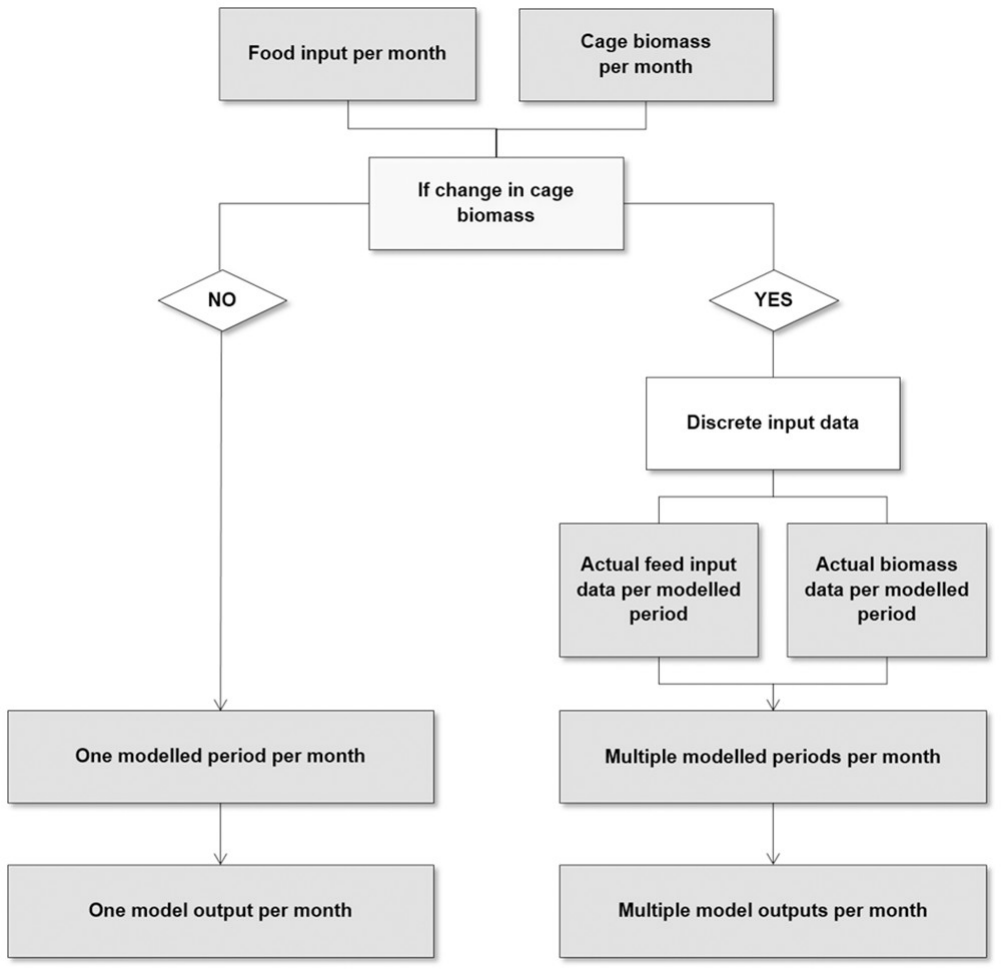
Flow diagram of the modifications in the process used to model waste dispersion per cage. Arrows indicate model outputs.

Month by month there were two possible approaches to modelling depending on the management practice for each cage. The approach used depended on whether the management practice influenced the production continuum, e.g. no change in fish biomass in the cage, or there was a change in cage management, e.g. there was a resultant change in fish biomass within the cage. The approaches are shown in Fig 3.

At intermittent intervals when cage biomass in fish cages changed, e.g. between October 2018 and July 2019, discrete periods of production were modelled for each fish cage per month. Under these circumstances, multiple modelled periods were established. Discrete input data accounted for variability in cage biomass, food types and quantities, settling velocities of food and faecal material, and cage dimensions and configurations, for every cage per month during the production period (Table 1). Cage management practices included:

- ‘cage input’ when fish batches were added to existing fish cages,
- ‘cage repositioning’ when existing fish cages were moved into new positions within the grid layout,
- ‘cage splitting’ that split fish batches in existing cages into multiple fish batches,
- ‘cage joining’ that combined fish batches from different cages,
- ‘site transfer’ when fish batches or cages were moved from the nursery facility to the offshore site for grow-out, and
- ‘cage harvesting’ (partial or complete).

**Table 1:** Cage production (cage biomass and feed inputs) and management practices at the fish farm between October 2018 and July 2019.

Cage No.	Species	Start Date	End Date	Opening Biomass (kg)	Closing Biomass (kg)	Period Transfer (-) (kg)	Period Transfer (+) (kg)	Period Stocking (kg)	Cage Move From	Cage Move To	Transaction Type	Net Size	Actual Feed Input (kg)	Feed Type
**October 2018**														
1	*S*. *aurata*	30/09/2018	31/10/2018	2705.69	4425.84							SS	2515	Pellet 1.5mm, Pellet 2.0mm
2	*S*. *aurata*	30/09/2018	31/10/2018	12567.59	16517.32							SS	6050	Pellet 3.0mm, Pellet 2.0mm
3	*S*. *dumerili*	30/09/2018	31/10/2018	172652.68	172924.00							MS	1800	Baitfish
4	*S*. *aurata*	30/09/2018	31/10/2018	28685.82	34034.93							MS	11968.75	Pellet 3.0mm, Pellet 2.0mm
5	*S*. *aurata*	23/10/2018	31/10/2018	0.00	58948.73		57890.1				Cage splitting	MS	4531.25	Pellet 4.5mm
	*S*. *aurata*	30/09/2018	22/10/2018	10296.79	0.00	-13971			5	7	Cage repositioning	SS	4306.25	Pellet 3.0mm, Pellet 2.0mm
6	*S*. *aurata*	30/09/2018	22/10/2018	110864.52	115765.67	-57890.1			6	5	Cage splitting	MS	18125	Pellet 4.5mm
	*S*. *aurata*	23/10/2018	31/10/2018	57967.35	58527.52							MS	1875	Pellet 4.5mm
7	*S*. *aurata*	22/10/2018	31/10/2018	0.00	14652.31		13971				Cage repositioning	SS	1718.75	Pellet 3.0mm
9	*S*. *aurata*	30/10/2018	31/10/2018	0.00	974.71			917.5			Cage input	SS	70	Pellet 1.0mm
10	*S*. *aurata*	30/09/2018	31/10/2018	4933.41	7041.65							SS	3000	Pellet 1.5mm, Pellet 2.0mm
11	*S*. *aurata*	30/09/2018	31/10/2018	4259.02	6326.85							SS	3000	Pellet 1.5mm, Pellet 2.0mm
12	*S*. *aurata*	30/10/2018	31/10/2018	0.00	875.99			817.5			Cage input	SS	70	Pellet 1.0mm
13	*S*. *aurata*	30/09/2018	31/10/2018	5250.72	7821.24							SS	3750	Pellet 2.0mm, Pellet 1.5mm
14	*S*. *aurata*	30/09/2018	31/10/2018	6831.02	8736.69							SS	3500	Pellet 2.0mm, Pellet 1.5mm
15	*S*. *aurata*	30/09/2018	31/10/2018	9258.46	11617.93							SS	4893.75	Pellet 2.0mm, Pellet 3.0mm
16	*S*. *aurata*	30/09/2018	31/10/2018	10922.82	14137.70							SS	4556.25	Pellet 3.0mm, Pellet 2.0mm
17	*S*. *aurata*	30/09/2018	31/10/2018	12593.49	13140.08							SS	4868.75	Pellet 3.0mm, Pellet 2.0mm
18	*S*. *aurata*	30/09/2018	31/10/2018	8323.95	12420.13							SS	4868.75	Pellet 2.0mm, Pellet 3.0mm
19	*S*. *aurata*	30/09/2018	31/10/2018	2608.93	4337.34							SS	2515	Pellet 1.5mm, Pellet 2.0mm
20	*S*. *aurata*	30/09/2018	31/10/2018	73324.19	81120.75							MS	17187.5	Pellet 3.0mm, Pellet 4.5mm
**November 2018**														
1	*S*. *aurata*	31/10/2018	30/11/2018	4425.84	5358.55							SS	2235	Pellet 2.0mm, Pellet 1.5mm
2	*S*. *aurata*	21/11/2018	30/11/2018	34866.85	36098.80		16508.9				Cage joining	MS	2656.3	Pellet 3.0mm
	*S*. *aurata*	31/10/2018	20/11/2018	16517.32	18357.85							MS	7656.25	Pellet 3.0mm
3	*S*. *dumerili*	31/10/2018	30/11/2018	172924.00	173348.51							MS	2300	Baitfish
4	*S*. *aurata*	31/10/2018	30/11/2018	34034.93	44581.13							MS	9593.75	Pellet 2.0mm, Pellet 3.0mm
5	*S*. *aurata*	31/10/2018	30/11/2018	58948.73	62989.13							MS	14218.75	Pellet 4.5mm, Pellet 4.5mm
6	*S*. *aurata*	31/10/2018	30/11/2018	58527.52	60399.06							MS	6406.25	Pellet 4.5mm, Pellet 4.5mm
7	*S*. *aurata*	22/11/2018	30/11/2018	0.00	1512.60			1244.92			Cage input	SS	350	Pellet 1.0mm, Pellet 1.5mm
	*S*. *aurata*	31/10/2018	21/11/2018	14652.31	0.00	-16508.86			7	2	Cage joining	SS	4062.5	Pellet 3.0mm
8	*S*. *aurata*	13/11/2018	30/11/2018	0.00	2456.05			1622.27			Cage input	SS	690	Pellet 1.5mm
9	*S*. *aurata*	31/10/2018	30/11/2018	974.71	2028.15							SS	1290	Pellet 1.0mm, Pellet 1.5mm
10	*S*. *aurata*	31/10/2018	30/11/2018	7041.65	10206.58							SS	2800	Pellet 2.0mm
11	*S*. *aurata*	31/10/2018	30/11/2018	6326.85	8704.89							SS	2775	Pellet 2.0mm
12	*S*. *aurata*	31/10/2018	30/11/2018	875.99	1939.38							SS	1290	Pellet 1.0mm, Pellet 1.5mm
13	*S*. *aurata*	31/10/2018	30/11/2018	7821.24	10512.57							SS	3812.5	Pellet 2.0mm, Pellet 3.0mm
14	*S*. *aurata*	31/10/2018	30/11/2018	8736.69	10913.41							SS	3812.5	Pellet 2.0mm, Pellet 3.0mm
15	*S*. *aurata*	31/10/2018	30/11/2018	11617.93	13056.44							SS	4026.875	Pellet 3.0mm, Pellet 2.0mm, Pellet 1.5mm
16	*S*. *aurata*	31/10/2018	30/11/2018	14137.70	16124.32							SS	4687.5	Pellet 3.0mm
17	*S*. *aurata*	31/10/2018	30/11/2018	13140.08	15100.52							SS	4687.5	Pellet 3.0mm
18	*S*. *aurata*	31/10/2018	30/11/2018	12420.13	14063.36							SS	4046.875	Pellet 3.0mm, Pellet 2.0mm
19	*S*. *aurata*	31/10/2018	30/11/2018	4337.34	5189.90							SS	2235	Pellet 2.0mm, Pellet 1.5mm
20	*S*. *aurata*	31/10/2018	30/11/2018	81120.75	87067.34							MS	12656.25	Pellet 3.0mm
**December 2018**														
1	*S*. *aurata*	30/11/2018	31/12/2018	5358.55	7031.89							SS	3587.5	Pellet 2.0mm
2	*S*. *aurata*	30/11/2018	31/12/2018	36098.80	40223.83							MS	11250	Pellet 3.0mm
3	*S*. *dumerili*	30/11/2018	31/12/2018	173348.51	173945.01							MS	4950	Baitfish
4	*S*. *aurata*	30/11/2018	31/12/2018	44581.13	47998.87							MS	10781.25	Pellet 3.0mm
5	*S*. *aurata*	30/11/2018	31/12/2018	62989.13	66395.41							MS	9843.75	Pellet 4.5mm, Pellet 4.5mm
6	*S*. *aurata*	30/11/2018	31/12/2018	60399.06	62011.05							MS	7343.75	Pellet 4.5mm, Pellet 4.5mm
7	*S*. *aurata*	30/11/2018	31/12/2018	1512.60	2261.90							SS	1305	Pellet 1.5mm, Pellet 1.0mm, Pellet 2.0mm
8	*S*. *aurata*	30/11/2018	31/12/2018	2456.05	3276.06							SS	1620	Pellet 1.5mm, Pellet 2.0mm
9	*S*. *aurata*	30/11/2018	31/12/2018	2028.15	3161.72							SS	1765	Pellet 1.5mm, Pellet 1.0mm, Pellet 2.0mm
10	*S*. *aurata*	30/11/2018	31/12/2018	10206.58	11609.16							SS	4125	Pellet 2.0mm
11	*S*. *aurata*	30/11/2018	21/12/2018	8704.89	0.00	-9472.24			11	20	Cage repositioning	SS	2175	Pellet 2.0mm
12	*S*. *aurata*	30/11/2018	31/12/2018	1939.38	2938.49							SS	1755	Pellet 1.5mm, Pellet 1.0mm, Pellet 2.0mm
13	*S*. *aurata*	30/11/2018	31/12/2018	10512.57	12023.08							SS	4512.5	Pellet 2.0mm, Pellet 3.0mm
14	*S*. *aurata*	30/11/2018	31/12/2018	10913.41	12298.48							SS	4337.5	Pellet 2.0mm, Pellet 3.0mm
15	*S*. *aurata*	30/11/2018	31/12/2018	13056.44	14598.32							SS	5512.5	Pellet 2.0mm, Pellet 3.0mm
16	*S*. *aurata*	30/11/2018	31/12/2018	16124.32	19454.78							SS	5546.88	Pellet 3.0mm
17	*S*. *aurata*	30/11/2018	31/12/2018	15100.52	23429.16							SS	5546.88	Pellet 3.0mm
18	*S*. *aurata*	30/11/2018	31/12/2018	14063.36	15587.15							SS	5512.5	Pellet 2.0mm, Pellet 3.0mm
19	*S*. *aurata*	30/11/2018	31/12/2018	5189.90	6521.87							SS	3137.5	Pellet 2.0mm
20	*S*. *aurata*	30/11/2018	20/12/2018	87067.34	0.00	-91283.88			20	M30	Site transfer	MS	8593.75	Pellet 3.0mm, Pellet 4.5mm, Pellet 4.5mm
	*S*. *aurata*	21/12/2018	31/12/2018	0.00	9845.00		9472.24				Cage repositioning	SS	1350	Pellet 2.0mm
**January 2019**														
1	*S*. *aurata*	31/12/2018	31/01/2019	7031.89	6.22							SS	2940.00	Pellet 2.0mm, Pellet 3.0mm, Pellet 1.0mm
2	*S*. *aurata*	31/12/2018	31/01/2019	40223.83	6.53							MS	8125.00	Pellet 3.0mm
3	*S*. *dumerili*	31/12/2018	31/01/2019	173945.01	28.25							MS	5100.00	Baitfish
4	*S*. *aurata*	31/12/2018	31/01/2019	47998.87	7.80							MS	8125.00	Pellet 3.0mm
5	*S*. *aurata*	31/12/2018	31/01/2019	66395.41	10.78							MS	6843.75	Pellet 4.5mm, Pellet 4.5mm
6	*S*. *aurata*	31/12/2018	31/01/2019	62011.05	10.07							MS	6818.75	Pellet 4.5mm, Pellet 4.5mm
7	*S*. *aurata*	31/12/2018	31/01/2019	2261.89	2.00							SS	1300.00	Pellet 2.0mm, Pellet 1.0mm, Pellet 1.5mm
8	*S*. *aurata*	31/12/2018	31/01/2019	3276.06	2.90							SS	1395.00	Pellet 2.0mm, Pellet 1.0mm, Pellet 1.5mm
9	*S*. *aurata*	31/12/2018	31/01/2019	3161.72	2.80							SS	1365.00	Pellet 1.0mm, Pellet 2.0mm, Pellet 1.5mm
10	*S*. *aurata*	31/12/2018	31/01/2019	11609.16	10.26							SS	3814.07	Pellet 2.0mm, Pellet 3.0mm, Pellet 3.0mm
12	*S*. *aurata*	31/12/2018	31/01/2019	2938.49	2.60							SS	1365.00	Pellet 2.0mm, Pellet 1.0mm, Pellet 1.5mm
13	*S*. *aurata*	31/12/2018	31/01/2019	12023.08	10.63							SS	4425.00	Pellet 3.0mm, Pellet 3.0mm, Pellet 2.0mm
14	*S*. *aurata*	31/12/2018	31/01/2019	12298.48	10.87							SS	4353.13	Pellet 3.0mm, Pellet 3.0mm, Pellet 2.0mm
15	*S*. *aurata*	31/12/2018	31/01/2019	14598.32	12.91							SS	4765.63	Pellet 3.0mm
16	*S*. *aurata*	31/12/2018	31/01/2019	19454.78	17.20							SS	4843.75	Pellet 3.0mm
17	*S*. *aurata*	31/12/2018	31/01/2019	23429.16	20.72							SS	4843.75	Pellet 3.0mm
18	*S*. *aurata*	31/12/2018	31/01/2019	15587.15	13.78							SS	4765.63	Pellet 3.0mm
19	*S*. *aurata*	31/12/2018	31/01/2019	6521.87	5.77							SS	2890.00	Pellet 2.0mm, Pellet 3.0mm, Pellet 1.0mm
20	*S*. *aurata*	31/12/2018	31/01/2019	9845.00	8.70							SS	3664.06	Pellet 2.0mm, Pellet 3.0mm, Pellet 3.0mm
**February 2019**														
1	*S*. *aurata*	31/01/2019	28/02/2019	8609.05	8944.45							SS	2175	Pellet 2.0mm, Pellet 2.0mm
2	*S*. *aurata*	31/01/2019	28/02/2019	53977.91	56377.17							MS	7812.5	Pellet 3.0mm
3	*S*. *dumerili*	31/01/2019	07/02/2019	174310.23	174368.78	-165682.1			3	M35	Site transfer	MS	3300	Baitfish
	*S*. *dumerili*	08/02/2019	28/02/2019	8685.07	8813.06								2200	Baitfish
4	*S*. *aurata*	31/01/2019	28/02/2019	51211.99	54638.44							MS	7933.75	Pellet 3.0mm
5	*S*. *aurata*	31/01/2019	12/02/2019	79772.68	80156.35							MS	11406.25	Pellet 4.5mm, Pellet 4.5mm
	*S*. *aurata*	13/02/2019	28/02/2019	161094.00	162533.18		80938.1				Cage joining	MS	8750	Pellet 4.5mm, Pellet 4.5mm
6	*S*. *aurata*	31/01/2019	13/02/2019	80538.31	0.00	-80938.12			6	5	Cage joining	MS	2656.25	Pellet 4.5mm, Pellet 4.5mm
	*S*. *aurata*	14/02/2019	28/02/2019	0.00	45304.80		43691.8				Cage joining	MS	4062.5	Pellet 3.0mm
7	*S*. *aurata*	31/01/2019	28/02/2019	2840.41	2968.18							SS	1205	Pellet 1.5mm, Pellet 2.0mm, Pellet 2.0mm
8	*S*. *aurata*	31/01/2019	28/02/2019	3832.24	5238.80							SS	1390	Pellet 2.0mm, Pellet 1.5mm, Pellet 2.0mm
9	*S*. *aurata*	31/01/2019	28/02/2019	3765.62	5449.78							SS	1230	Pellet 1.5mm, Pellet 2.0mm, Pellet 2.0mm
10	*S*. *aurata*	31/01/2019	28/02/2019	12603.01	17455.73							SS	2915.63	Pellet 3.0mm, Pellet 2.0mm, Pellet 2.0mm
12	*S*. *aurata*	31/01/2019	18/02/2019	3554.11	0.00	-4775.72			12	15	Cage repositioning	SS	730	Pellet 1.5mm, Pellet 2.0mm
13	*S*. *aurata*	31/01/2019	28/02/2019	13107.43	13537.08							SS	3675	Pellet 2.0mm, Pellet 3.0mm, Pellet 2.0mm
14	*S*. *aurata*	31/01/2019	28/02/2019	17230.47	18204.57							SS	3906.25	Pellet 3.0mm
15	*S*. *aurata*	31/01/2019	14/02/2019	19406.84	0.00	-20192.70			15	6	Cage joining	SS	2031.25	Pellet 3.0mm
	*S*. *aurata*	18/02/2019	28/02/2019	0.00	4943.94		4775.72				Cage repositioning	SS	520	Pellet 1.5mm, Pellet 2.0mm, Pellet 2.0mm
16	*S*. *aurata*	31/01/2019	06/02/2019	20844.95	0.00	-21245.04			16	20	Cage joining	SS	781.25	Pellet 3.0mm
17	*S*. *aurata*	31/01/2019	04/02/2019	25623.03	0.00	-25822.02			17	20	Cage repositioning	SS	468.75	Pellet 3.0mm
	*S*. *aurata*	04/02/2019	28/02/2019	0.00	15406.56		10891.1				Cage repositioning	SS	2621.872	Pellet 3.0mm, Pellet 2.0mm, Pellet 2.0mm
18	*S*. *aurata*	31/01/2019	14/02/2019	22957.38	0.00	-23499.11			18	6	Cage joining	SS	2031.25	Pellet 3.0mm
19	*S*. *aurata*	31/01/2019	28/02/2019	7776.73	8230.36							SS	2200	Pellet 2.0mm, Pellet 2.0mm
20	*S*. *aurata*	04/02/2019	28/02/2019	0.00	49577.73		47067.1				Cage joining	MS	6996.874	Pellet 3.0mm, Pellet 2.0mm
	*S*. *aurata*	31/01/2019	04/02/2019	10810.25	0.00	-10891.09			20	17	Cage repositioning	SS	328.124	Pellet 3.0mm, Pellet 2.0mm
**March 2019**														
1	*S*. *aurata*	15/03/2019	31/03/2019	0.00	34355.99		33436.6				Cage joining	MS	3656.25	Pellet 2.0mm, Pellet 3.0mm, Pellet 2.0mm
	*S*. *aurata*	28/02/2019	15/03/2019	8944.45	0.00	-12280.70			1	17	Cage repositioning	SS	1250	Pellet 2.0mm, Pellet 2.0mm
2	*S*. *aurata*	28/02/2019	31/03/2019	56377.17	62957.83							MS	9531.25	Pellet 3.0mm
3	*S*. *dumerili*	28/02/2019	31/03/2019	8813.06	9170.73							SS	4100	Baitfish
4	*S*. *aurata*	28/02/2019	31/03/2019	54638.44	63702.38							SS	9531.25	Pellet 3.0mm
5	*S*. *aurata*	28/02/2019	31/03/2019	162533.18	164980.16							MS	15625	Pellet 4.5mm, Pellet 4.5mm
6	*S*. *aurata*	28/02/2019	31/03/2019	45304.80	48830.88				6	19	Cage joining	MS	7578.125	Pellet 3.0mm
7	*S*. *aurata*	28/02/2019	31/03/2019	2968.18	5012.26							SS	1660	Pellet 1.5mm, Pellet 2.0mm, Pellet 2.0mm
8	*S*. *aurata*	28/02/2019	19/03/2019	5238.80	0.00	-5487.83			8	16	Cage repositioning	SS	850	Pellet 2.0mm, Pellet 2.0mm
9	*S*. *aurata*	28/02/2019	31/03/2019	5449.78	6137.14							SS	1700	Pellet 2.0mm, Pellet 2.0mm
10	*S*. *aurata*	28/02/2019	15/03/2019	17455.73	17728.50	-17728.50			10	1	Cage joining	SS	1150	Pellet 2.0mm, Pellet 2.0mm
13	*S*. *aurata*	28/02/2019	26/03/2019	13537.08	0.00	-16864.15			13	19	Cage joining	SS	3515.625	Pellet 3.0mm
14	*S*. *aurata*	28/02/2019	25/03/2019	18204.57	0.00	-19056.88			14	19	Cage repositioning	SS	3359.375	Pellet 3.0mm
15	*S*. *aurata*	28/02/2019	31/03/2019	4943.94	5651.60							SS	1745	Pellet 2.0mm, Pellet 1.5mm, Pellet 2.0mm
16	*S*. *aurata*	19/03/2019	31/03/2019	0.00	5881.55		5487.83				Cage repositioning	SS	750	Pellet 2.0mm, Pellet 2.0mm
17	*S*. *aurata*	28/02/2019	15/03/2019	15406.56	0.00	-15708.10			17	1	Cage joining	SS	1225	Pellet 2.0mm, Pellet 2.0mm
	*S*. *aurata*	15/03/2019	31/03/2019	0.00	12772.87		12280.7				Cage repositioning	SS	2196.875	Pellet 2.0mm, Pellet 3.0mm
18	*S*. *aurata*	25/03/2019	31/03/2019	0.00	12255.21		12033.2				Cage repositioning	SS	1093.75	Pellet 3.0mm
19	*S*. *aurata*	26/03/2019	31/03/2019	0.00	36245.81		359210				Cage joining	MS	2031.25	Pellet 3.0mm
	*S*. *aurata*	28/02/2019	25/03/2019	8230.36	0.00	-12033.24			19	18	Cage repositioning	SS	2275	Pellet 2.0mm, Pellet 2.0mm, Pellet 3.0mm
20	*S*. *aurata*	28/02/2019	31/03/2019	49577.73	53827.94							SS	9218.75	Pellet 3.0mm
**April 2019**														
1	*S*. *aurata*	31/03/2019	30/04/2019	34355.99	36271.91							MS	6825	Pellet 3.0mm, Pellet 2.0mm
2	*S*. *aurata*	31/03/2019	30/04/2019	62957.83	66131.56							MS	10781.25	Pellet 3.0mm, Pellet 4.5mm, Pellet 4.5mm
3	*S*. *dumerili*	31/03/2019	30/04/2019	9170.73	9371.50							MS	4560	Baitfish
4	*S*. *aurata*	31/03/2019	30/04/2019	63702.38	67964.38							MS	8750	Pellet 3.0mm
5	*S*. *aurata*	31/03/2019	30/04/2019	164980.16	167258.50							MS	14531.25	Pellet 4.5mm, Pellet 4.5mm, Pellet 3.0mm
6	*S*. *aurata*	31/03/2019	30/04/2019	48830.88	52618.13							MS	7443.75	Pellet 3.0mm, Pellet 2.0mm
7	*S*. *aurata*	31/03/2019	30/04/2019	5012.26	5855.65							SS	2100	Pellet 2.0mm, Pellet 2.0mm
8	*S*. *aurata*	30/04/2019	30/04/2019	0.00	781.47			772.50			Cage input	SS	20	Pellet 1.5mm, Pellet 1.0mm
9	*S*. *aurata*	31/03/2019	30/04/2019	6137.14	9841.42							SS	2125	Pellet 2.0mm, Pellet 2.0mm
10	*S*. *aurata*	09/04/2019	30/04/2019	0.00	581.30			378.978			Cage input	SS	420	Pellet 1.5mm, Pellet 1.0mm
11	*S*. *aurata*	09/04/2019	30/04/2019	0.00	563.67			366.94			Cage input	SS	420	Pellet 1.5mm, Pellet 1.0mm
13	*D*. *labrax*	11/04/2019	30/04/2019	0.00	1482.95			1038.87			Cage input	SS	830	Pellet 2.0mm, Pellet 2.0mm, Pellet 1.5mm
14	*D*. *labrax*	13/04/2019	30/04/2019	0.00	1382.15			981.96			Cage input	SS	730	Pellet 2.0mm, Pellet 1.5mm
15	*S*. *aurata*	31/03/2019	30/04/2019	5651.60	8089.11							SS	2125	Pellet 2.0mm, Pellet 2.0mm
16	*S*. *aurata*	31/03/2019	30/04/2019	5881.55	6306.84							SS	2025	Pellet 2.0mm, Pellet 2.0mm
17	*S*. *aurata*	31/03/2019	16/04/2019	12772.87	0.00	-13285.49			17	18	Cage joining	SS	2187.5	Pellet 3.0mm
	*S*. *aurata*	30/04/2019	30/04/2019	0.00	781.59			772.5			Cage input	SS	20	Pellet 1.5mm, Pellet 1.0mm
18	*S*. *aurata*	16/04/2019	30/04/2019	26093.00	26901.84		13285.5				Cage joining	MS	2837.5	Pellet 3.0mm, Pellet 2.0mm, Pellet 2.0mm
	*S*. *aurata*	31/03/2019	15/04/2019	12255.21	12808.90							MS	5025	Pellet 3.0mm, Pellet 2.0mm, Pellet 2.0mm
19	*S*. *aurata*	31/03/2019	30/04/2019	36245.81	38170.66							MS	7131.25	Pellet 3.0mm, Pellet 2.0mm
20	*S*. *aurata*	31/03/2019	30/04/2019	53827.94	57699.95							MS	9062.5	Pellet 3.0mm, Pellet 4.5mm, Pellet 4.5mm
**May 2019**														
1	*S*. *aurata*	30/04/2019	15/05/2019	36271.91	0.00	-37706.51			1	M23	Site transfer	MS	4575	Pellet 3.0mm, Pellet 2.0mm
2	*S*. *aurata*	17/05/2019	31/05/2019	535.35	744.41			535.36			Cage input	SS	330	Pellet 1.0mm, Pellet 1.5mm
	*S*. *aurata*	30/04/2019	14/05/2019	66131.56	0.00	-68603			2	M34	Site transfer	MS	5468.75	Pellet 4.5mm, Pellet 3.0mm
3	*S*. *dumerili*	02/05/2019	31/05/2019	3873.26	4270.89						Harvest	MS	4300	Baitfish
	*S*. *dumerili*	30/04/2019	01/05/2019	9371.50	9389.39						Harvest	MS	4900	Baitfish
4	*S*. *aurata*	30/04/2019	31/05/2019	67964.38	77528.02							MS	13693.75	Pellet 3.0mm, Pellet 2.0mm
5	*S*. *aurata*	30/04/2019	11/05/2019	167258.50	0.00	-181139.9			5	M35	Site transfer	SS	5000	Pellet 4.5mm
6	*S*. *aurata*	30/04/2019	31/05/2019	52618.13	61560.86							MS	11812.5	Pellet 3.0mm, Pellet 2.0mm
7	*S*. *aurata*	30/04/2019	31/05/2019	5855.65	6668.64							SS	2325	Pellet 2.0mm, Pellet 2.0mm
8	*S*. *aurata*	30/04/2019	31/05/2019	781.47	1527.60							SS	1010	Pellet 1.5mm, Pellet 1.0mm
9	*S*. *aurata*	30/04/2019	31/05/2019	9841.42	10838.66							SS	2950	Pellet 2.0mm, Pellet 2.0mm
10	*S*. *aurata*	30/04/2019	31/05/2019	581.30	1008.77							SS	710	Pellet 1.0mm, Pellet 1.5mm
11	*S*. *aurata*	30/04/2019	31/05/2019	563.67	987.56							SS	710	Pellet 1.5mm, Pellet 1.0mm
13	*D*. *labrax*	30/04/2019	31/05/2019	1482.95	2298.35							SS	1375	Pellet 1.5mm, Pellet 2.0mm
14	*D*. *labrax*	30/04/2019	31/05/2019	1382.15	2116.50							SS	1375	Pellet 2.0mm, Pellet 1.5mm
15	*S*. *aurata*	30/04/2019	31/05/2019	8089.11	9173.21							SS	2950	Pellet 2.0mm, Pellet 2.0mm
16	*S*. *aurata*	30/04/2019	31/05/2019	6306.84	6770.41							MS	2350	Pellet 2.0mm, Pellet 2.0mm
17	*S*. *aurata*	30/04/2019	31/05/2019	781.59	1561.57							SS	1010	Pellet 1.5mm, Pellet 1.0mm
18	*S*. *aurata*	30/04/2019	31/05/2019	26901.84	28425.11							MS	8437.5	Pellet 3.0mm, Pellet 2.0mm
19	*S*. *aurata*	30/04/2019	31/05/2019	38170.66	47133.49							MS	10325	Pellet 2.0mm, Pellet 3.0mm
20	*S*. *aurata*	17/05/2019	31/05/2019	535.35	745.30			535.36			Cage input	SS	330	Pellet 1.0mm, Pellet 1.5mm
	*S*. *aurata*	30/04/2019	11/05/2019	57699.95	0.00	-59254.43			20	M36	Site transfer	MS	3750	Pellet 3.0mm, Pellet 4.5mm
**June 2019**														
1	*S*. *aurata*	07/06/2019	30/06/2019	0.00	8554.35		7417.73				Cage repositioning	SS	3325	Pellet 2.0mm
2	*S*. *aurata*	31/05/2019	30/06/2019	744.41	1758.21							SS	1220	Pellet 1.0mm, Pellet 1.5mm
3	*S*. *dumerili*	31/05/2019	30/06/2019	4270.89	4677.74							MS	3300	Baitfish
4	*S*. *aurata*	03/06/2019	30/06/2019	0.00	23126.63		20148.4				Cage joining	MS	8525	Pellet 2.0mm, Pellet 3.0mm
5	*S*. *aurata*	14/06/2019	30/06/2019	0.00	701.53			369.90			Cage input	SS	425	Pellet 1.0mm, Pellet 1.5mm
6	*S*. *aurata*	31/05/2019	30/06/2019	61560.86	70611.11							MS	19843.75	Pellet 3.0mm, Pellet 4.5mm
7	*S*. *aurata*	31/05/2019	30/06/2019	6668.64	7323.75							SS	3925	Pellet 2.0mm
8	*S*. *aurata*	31/05/2019	30/06/2019	1527.60	2372.44							SS	1995	Pellet 1.5mm, Pellet 2.0mm
9	*S*. *aurata*	31/05/2019	03/06/2019	10838.66	0.00	-10911.02			9	4	Cage joining	SS	200	Pellet 2.0mm
10	*S*. *aurata*	31/05/2019	30/06/2019	1008.77	1730.68							SS	1170	Pellet 1.5mm, Pellet 1.0mm
11	*S*. *aurata*	31/05/2019	30/06/2019	987.56	1473.00							SS	1200	Pellet 1.5mm, Pellet 1.0mm
13	*D*. *labrax*	31/05/2019	30/06/2019	2298.35	4293.40							SS	2632.5	Pellet 2.0mm, Pellet 1.5mm
14	*D*. *labrax*	31/05/2019	30/06/2019	2116.50	4058.26							SS	2632.5	Pellet 1.5mm, Pellet 2.0mm
15	*S*. *aurata*	14/06/2019	30/06/2019	0.00	663.28			369.90			Cage input	SS	425	Pellet 1.0mm, Pellet 1.5mm
	*S*. *aurata*	31/05/2019	03/06/2019	9173.21	0.00	-9237.38			15	4	Cage joining	SS	200	Pellet 2.0mm
16	*S*. *aurata*	31/05/2019	07/06/2019	6770.41	0.00	-7417.73			16	1	Cage repositioning	SS	600	Pellet 2.0mm
17	*S*. *aurata*	31/05/2019	30/06/2019	1561.57	2277.31							SS	1995	Pellet 1.5mm, Pellet 2.0mm
18	*S*. *aurata*	31/05/2019	30/06/2019	28425.11	33926.85							MS	11562.5	Pellet 3.0mm
19	*S*. *aurata*	31/05/2019	30/06/2019	47133.49	53362.40							MS	14881.25	Pellet 3.0mm, Pellet 2.0mm
20	*S*. *aurata*	31/05/2019	30/06/2019	745.30	1770.54							SS	1220	Pellet 1.0mm, Pellet 1.5mm
**July 2019**														
1	*S*. *aurata*	30/06/2019	31/07/2019	8554.35	11169.16							SS	6575	Pellet 2.0mm, Pellet 3.0mm
2	*D*. *labrax*	12/07/2019	31/07/2019	0.00	15048.41		10015.3				Cage joining	MS	6846.875	Pellet 2.0mm, Pellet 3.0mm
	*D*. *labrax*	30/06/2019	11/07/2019	1758.21	0.00	-2305.16			2	19	Cage repositioning	SS	795	Pellet 2.0mm, Pellet 1.5mm, Pellet 1.0mm
3	*S*. *dumerili*	30/06/2019	14/07/2019	4677.74	4841.07						Harvest	MS	2900	Baitfish
	*S*. *dumerili*	15/07/2019	18/07/2019	2800.69	2838.71						Harvest	MS	200	Baitfish
	*S*. *dumerili*	19/07/2019	31/07/2019	2689.40	2781.25						Harvest	MS	800	Baitfish
4	*S*. *aurata*	30/06/2019	31/07/2019	23126.63	28406.69							MS	12506.25	Pellet 2.0mm, Pellet 3.0mm
5	*S*. *aurata*	30/06/2019	31/07/2019	701.53	2073.23							SS	1770	Pellet 1.0mm, Pellet 1.5mm
6	*S*. *aurata*	30/06/2019	31/07/2019	70611.11	77283.26							MS	19531.25	Pellet 3.0mm, Pellet 4.5mm
7	*S*. *aurata*	30/06/2019	31/07/2019	7323.75	10228.85							SS	6121.88	Pellet 2.0mm, Pellet 3.0mm
8	*S*. *aurata*	30/06/2019	31/07/2019	2372.44	4854.06							SS	3730	Pellet 1.5mm, Pellet 2.0mm, Pellet 1.0mm
10	*S*. *aurata*	30/06/2019	31/07/2019	1730.68	3321.85							SS	2407.5	Pellet 1.5mm, Pellet 2.0mm, Pellet 1.0mm
11	*S*. *aurata*	30/06/2019	31/07/2019	1473.00	3111.44							SS	2502.5	Pellet 2.0mm, Pellet 1.5mm, Pellet 1.0mm, Pellet 1.5mm
12	*S*. *aurata*	03/07/2019	31/07/2019	0.00	1816.31			532.5			Cage input	SS	1645	Pellet 1.0mm, Pellet 1.5mm, Pellet 1.0mm, Pellet 1.5mm
13	*D*. *labrax*	30/06/2019	12/07/2019	4293.40	0.00	-5104.70			13	2	Cage joining	SS	1175	Pellet 2.0mm
14	*D*. *labrax*	30/06/2019	12/07/2019	4058.26	0.00	-4910.60			14	2	Cage joining	SS	1225	Pellet 2.0mm
15	*S*. *aurata*	30/06/2019	31/07/2019	663.28	2073.66							SS	1750	Pellet 1.0mm, Pellet 1.5mm, Pellet 1.0mm, Pellet 1.5mm
16	*S*. *aurata*	03/07/2019	31/07/2019	532.50	1813.02			532.50			Cage input	SS	1620	Pellet 1.0mm, Pellet 1.5mm
17	*S*. *aurata*	30/06/2019	31/07/2019	2277.31	4810.21							SS	3830	Pellet 1.5mm, Pellet 2.0mm, Pellet 1.0mm, Pellet 1.0mm, Pellet 1.5mm
18	*S*. *aurata*	30/06/2019	31/07/2019	33926.85	45437.23							MS	17031.25	Pellet 3.0mm, Pellet 4.5mm
19	*S*. *aurata*	11/07/2019	31/07/2019	2305.16	3678.22		2305.16		2	19	Cage repositioning	SS	2027.5	Pellet 2.0mm, Pellet 1.5mm
	*S*. *aurata*	30/06/2019	11/07/2019	53362.40	0.00	-55225.01			19	M27	Site transfer	MS	5000	Pellet 3.0mm
20	*S*. *aurata*	30/06/2019	31/07/2019	1770.54	3678.96							SS	2822.5	Pellet 1.5mm, Pellet 2.0mm, Pellet 1.0mm

SS indicates 12 m diameter fish cages, MS indicates 28 m diameter fish cages

When cage management practices influenced cage biomass in fish cages at the fish farm, discrete input data was modelled instead of monthly food and cage biomass information. The consequent changes in fish farm cage positioning were accounted for by repositioning grid cells for cages in the model. Fish farm management practices were identified in husbandry data provided by the farm manager and discrete periods of production were modelled when cage biomass was constant prior to and following intervals in production (Table 1). Distinctly different cage production setups were modelled separately. For instance, discrete periods of production were modelled separately around fish farm operations when the cage biomass was split or combined using detailed input data to improve the representativeness of fish farm production (see Fig 3).

Actual feed input data (Table 1) included 1 mm, 1.5 mm, 2 mm, 3 mm, and 4.5 mm feeds that were administered in succession or in combination at the fish farm. Generally, the extruded feeds that were supplemented to sea bream and sea bass included marine sources, terrestrial plant-based sources and the derivatives of terrestrial animals. In addition, thawed and chopped Atlantic mackerel (*Scomber scombrus*) was used as baitfish supplemented to amberjack at the fish farm.

Settlement velocities of formulated feeds used in sea bream and sea bass production in the study were deduced from literature (Table 2). The settling rate of baitfish used in amberjack production was based on estimates in [21]. The faecal pellet velocities used in the model were 0.005 m s^-1^ for sea bream and 0.007 m s^-1^ for sea bass [1]. Settling velocities were consistent for various faecal pellet sizes from different fish sizes [1, 22, 23]. The settling velocity for the faecal material of amberjack (0.005 m s^-1^) was assumed according to estimations for baitfish faeces in [21].

**Table 2:** Feed settling velocities for sea bream, sea bass and amberjack.

Feed type	Settling velocity (m/s)
1 mm feed pellet	0.04 ^a^^,^ ^b^
1.5 mm feed pellet	0.062 ^a^^,^ ^b^
2 mm feed pellet	0.079 ^a^^,^ ^b^
3 mm feed pellet	0.087 ^c^
4.5 mm feed pellet	0.103 ^c^
Chopped baitfish	0.07 ^d^

^a^ [22],

^b^ [4],

^c^ [24],

^d^ [21].

In the mass-balance model, default nutrient input parameters were defined for the production of sea bream, sea bass, and amberjack, according to literature, listed in Table 3. Values for nutrient uptake by fish were changed for the feed used (e.g. pellet size), the farmed species, and size of fish.

**Table 3:** Nutrient input parameters and assumptions for sea bream, sea bass and amberjack.

Parameter	Sea bream	Sea bass	Amberjack
Relative food wastage	33% ^a^	38% ^a^	38% ^b^
Carbon content of feed	48.6–50.1% ^c^	49.2–50.9% ^c^	46.6% ^c^
Carbon content of fish tissue	15% ^d^^,^ ^e^	15% ^d^^,^ ^e^	15% ^d^^,^ ^e^
Relative respired carbon	60% ^d^^,^ ^f^	60% ^d^^,^ ^f^	60% ^d^^,^ ^f^
Carbon content of faeces	36.7% ^c^	33.1% ^c^	36.7%

^a^ [27],

^b^ [25],

^c^ [28],

^d^ [26],

^e^ [22],

^f^ [29].

Assumptions were made as some species and feed-specific information was limited particularly for amberjack production. The wastage of baitfish in amberjack production was adopted from the estimated wastage of trash fish supplemented to the areolate grouper (*Epinephelus areolatus*) in open-sea cages elsewhere [25] (Table 3). General mass balance estimates for carbon in the food-fish-waste system were adopted from [22, 26]. The assumed respired fraction also accounts for losses through urea [26].

After mass balance estimations, the horizontal dispersion of waste within the model, prior to initial settlement, was based on hydrographic data, settlement velocities, water depth and horizontal distance dispersed from the cages. The overall particulate waste distribution on the seabed was modelled for the fish cages within the site, arranged individually on a grid system, as described in [19], adapted for 5 m grid resolution. The 12 m diameter fish cages were represented by five grid cells and the 28 m diameter fish cages represented by 25 cells in this study. For each discrete modelled period, grid cells were repositioned to reflect cage movements and configurations. The amount of particulate waste released by fish cages at the site was estimated as deposition of organic carbon in 5 x 5 m grid cells.

The final data outputs of the dispersion models for each cage, for each distinct modelled periods, were overlaid as layers of individual worksheets to form a single worksheet for every month. The combined data output was imported into Surfer 16 (Golden Software Inc., USA) to produce two-dimensional contour maps for a visual representation of waste dispersion around the fish farm per month.

No permits were required for the described study, which complied with all relevant regulations and access to fish farm facilities provided with the consent of the operator.

## Results

**Fig 4:**
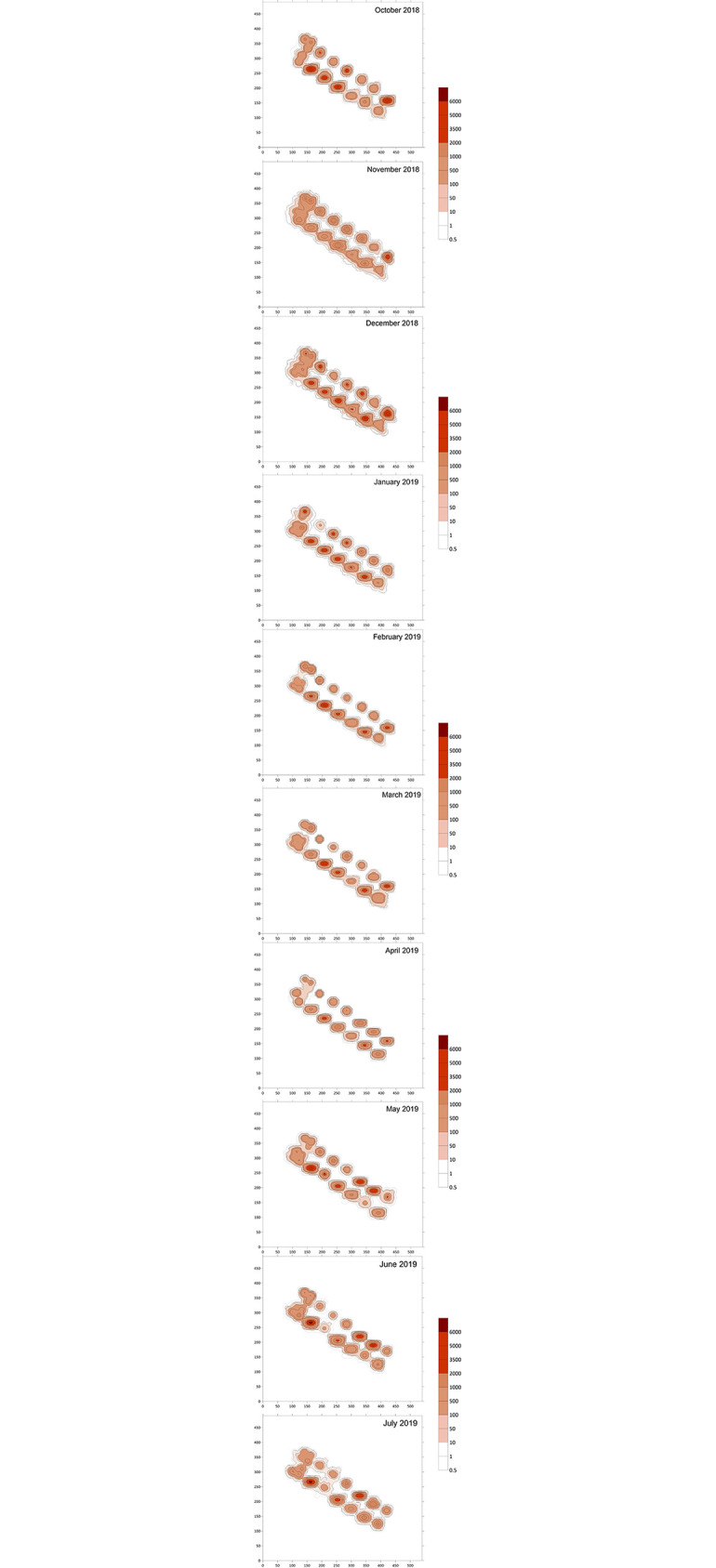
Particulate waste dispersion from fish in fish cages to the seabed on monthly basis over a twelve-month period (October 2018 to July 2019). Axis units are in metres North (Y-axis) and East (X-axis), and deposition contours are in total gC m^-2^ accumulation over the month.

Model estimates and patterns of sediment carbon (gC m^-2^) deposition near cages at the fish farm represent the spatial and temporal effects of cage management practices on waste dispersion. Model outputs produced as contour plots in Surfer^™^ are presented in Fig 4 for each month between October 2018 and July 2019.

The contours show the considerable variation in deposition of waste for the different cages, within each month modelled and between each month modelled. Model outputs also revealed higher deposition directly below the fish cages that was localised and decreased with increasing distance from the fish cages. Deposition rates and patterns exhibit variation that is presumably associated with alteration in cage biomass and food input, and modifications in cage sizes and arrangement throughout production, which are attributed to farm-level management practices (Table 1).

The deposition footprints and the maximum deposition rate, F_max_ (shown in Table 4 for each cage during each study month), reveal cage-level differences in deposition during the same periods. For instance, the deposition in June 2019 changed from a negligible F_max_ (>1 gCm^-2^) to the highest F_max_ (6852 gC m^-2^) recorded. In addition, the largest F_max_ depositional change is for Cage 6 showing a 5559 gC m^-2^ difference over the study period, with all cages showing a minimum of 57% change in their F_max_ value. Again, this illustrates the considerable variability of deposition of particulate waste over time and with change in farm practice. These changes have significant implications for variability in environmental conditions within the sediments over time for each cage, and for food availability for benthic organisms being grown near to the cages within an IMTA system.

**Table 4:** Maximum deposition rates (F_max_) predicted directly below fish cage positions every month between October 2018 and July 2019.

	F_max_ (gC m^-2^)									
Cage number	October	November	December	January	February	March	April	May	June	July
1	835.1	411.3	1332.3	1384.2	979.3	1881.4	1530.2	1778.8	1358.7	1513.4
2	1581.1	1311.3	4574.1	2877.7	2421.6	3981.3	2137.9	488.3	520.5	1788.1
3	796.6	1007.5	2087.1	2201.4	1367.4	630.9	629.2	1627.4	939.8	1005.8
4	3822.8	1824.3	4251.4	2858.7	2548.5	3516.2	1824.8	2694.0	2711.0	3398.7
5	2691.1	1026.3	3011.3	2842.7	4410.1	5254.1	2311.6	1992.5	117.8	431.1
6	5641.3	1299.7	3204.1	2835.8	2366.7	1995.0	1387.1	5898.3	6858.3	6148.4
7	746.9	944.2	100.4	543.1	529.3	779.3	615.3	1158.8	1633.1	1187.4
8	0.6	56.5	445.2	581.7	319.1	407.1	1.6	465.7	543.7	1162.3
9	13.9	101.2	337.1	549.9	287.1	802.6	695.2	1170.0	105.1	10.1
10	936.2	238.5	1508.4	1728.4	504.0	589.7	98.4	324.7	355.0	1011.2
11	948.5	553.7	877.2	10.4	0.6	5.4	98.6	273.8	352.8	796.8
12	14.7	449.5	396.1	428.8	50.9	1.9	10.3	0.3	0.2	409.8
13	1164.4	1163.5	1998.3	2178.4	517.1	1177.6	387.1	641.6	1011.2	356.3
14	1119.7	1282.2	1560.9	54.1	1589.4	1728.7	291.2	605.7	1006.3	370.0
15	1694.1	1338.6	2441.4	135.2	984.6	781.9	617.9	1179.4	299.1	459.2
16	816.6	1645.2	651.2	2051.3	598.8	322.9	762.2	1341.1	126.2	407.6
17	2355.3	1677.3	2004.9	2017.6	408.3	1747.5	917.4	465.1	543.3	1452.1
18	936.8	1442.7	2445.6	2000.7	813.7	572.9	1787.1	3287.3	3798.7	2646.0
19	823.1	597.7	1098.7	1289.5	913.8	1095.6	1385.7	3555.5	4098.8	1704.3
20	4934.5	3012.3	3337.6	1784.3	2463.1	3582.4	2018.6	2188.3	501.1	1012.1

## Discussion

This study illustrates some of the complexities in marine fish farming and the implications of real-world farming practices and reveals challenges for optimal placement of benthic species in IMTA systems. The results show that cohort dynamics and cage movements associated with the aquaculture management practices of the fish farm influenced the dispersion of wastes. Deposition footprints revealed variability that reflects the cage management complexities that affect the quantity and fate of wastes distributed around fish cages. Consequently, better representation of these farm-specific dynamics and practices can improve the accuracy and realism of farm-scale model estimations. This study shows that representative input data is needed to describe actual food inputs and cage biomass changes, and to account for cage management practices.

The predicted deposition around multiple species and cohorts reflects real-world aquaculture that does not follow a production continuum, but F_max_ calculation reveals variation driven by the multitude of husbandry practices described throughout production. The cage management practices described in this study are required to accommodate production demands. These practices result in multiple cohorts of different species and sizes of fish being stocked in adjacent cages on the same fish farm, and inevitably influence the standing biomass of farmed fish in fish cages and the feeding requirements at the fish farm. Moreover, these practices result in intermittent changes in the layout of the fish cages within the farm. While models do not always have the capability to adapt to different sites and production settings [30], when applied to this fish farm, the farm-scale model within this study, also resolved important spatial and temporal variability in sediment carbon enrichment. Model studies that considered different cohort and feed input data in individual cages at the same farm improved model accuracy [1, 4] whereas others applied to different aquaculture scenarios revealed that farm management practices were important criteria for better predictions [17, 31]. Where management practices complicate cage arrangements and change cage biomass and food inputs to divert production trends, effective monitoring and management of environmental impact need a finer assessment modelling approach to account for the variability revealed at cage level.

There are natural and anthropogenic dynamics that influence water movement with variation in currents within the vertical water gradient and consequently, can affect the distribution and fate of wastes [20]. Therefore, these dynamics need to be considered when trying to obtain a representative footprint for setting up of bottom dwelling IMTA. Detailed description of local currents in real time accounts for episodic events of severe weather and other real-world complications that are not necessarily represented by short-term or averaged hydrography data. Though there have been considerable advances in development and use of two- or three-dimensional hydrodynamic models to simulate aquaculture waste dispersion [32, 33], modelling complex coastal areas at sufficient resolution is still a challenge [34]. With any environmental sampling and modelling, there are limitations as neither models nor *in-situ* measurements can capture the full complexity of complex environments [35]. Still, there needs to be sufficient information to recognise the dynamic nature of the environment and represent the farm environment beyond oversimplistic generalisations. Decision-support models used in aquaculture planning and licensing need reliable data that capture the true extent and severity of impact as production changes with farm-level management decisions. Based on findings from the present study, it is recommended that waste dispersion models use hydrographic inputs, farm layouts that include changes over time, and simulate realistic and variable production practices. Since models can have an important role in aquaculture planning, licensing and regulation [36], it is important that models are realistic for environmental and production considerations, so that representative computations of deposition footprints are obtained, supporting production levels within capacity limits.

Waste dispersion models also have an important role to play in IMTA research and development as models are required to understand the potential nutrient transfer, environmental interactions and production consequences of co-cultivating species under different production scenarios [36, 37]. For a balanced system with net removal of wastes, models need to quantify and resolve the distribution and impact of particulate wastes around the fish farm [38, 39]. Resolving farm-level complexities can provide essential information about the variability of food availability and quality for extractive organisms to recycle aquaculture-derived organic wastes effectively and to maximise production. For example, understanding how the carbon deposition footprint changes helps producers to site and manage extractive organisms (e.g. deposit-feeding sea cucumbers) effectively near fish cages [39]. Although deposit-feeding sea cucumbers grow better on organically rich sediments [39–41], mass mortalities were recorded in sea cucumbers placed in an area of predicted high organic waste deposition directly below fish cages [39]. However, within a few metres from the fish cages, sea cucumbers survived and grew well [39]. Moreover, growth and physiological responses of sea cucumbers have been ascribed to the temporal variation in food quantity and quality in seafloor sediments under fish cages [42–45] to add further scope for finer resolution modelling in local, farm-scale IMTA application.

In practice, to optimise IMTA production, location of the deposit-feeding, extractive species should account for the variability in waste distribution associated with cage production and the effects on benthic conditions, so the physiological requirements of extractive species throughout its entire grow-out production can be adequately satisfied. Models such as the one used in this study, can be used to help identify appropriate placement of the extractive IMTA species, but only if they are representative of the site and fish cage production practices. Clearly, generalisations of site characteristics could under or over-estimate the amount of waste available for the extractive species with implications for survivability.

## Conclusion

Where multiple species, cohort dynamics and irregular cage arrangements influence production continuity in intensive cage production, variability in deposition footprints reveals cage-level complexities that need to be considered for improved model predictions. In this study, the farm-scale model resolved variation in the initial particulate waste settlement as a function of cage-level considerations. This finer modelling approach towards predicting waste distribution and benthic impact is down to detailed input data and farm-specific considerations for cage level variability. For licensing and environmental regulation, predicting the magnitude of the deposition footprint and the influence of cage management practices can contribute towards more representative assessments and effective management of benthic impacts. While environmental impact assessments and monitoring efforts are typically staggered one-time or one-point occasions, cage production is variable and even if different model scenarios are considered, in practice real-world complexities need to be accounted for effective management. Then, to mitigate benthic impacts efficiently through IMTA, the availability and quality of particulate organic wastes in seafloor sediments during cage production needs detailed representation. The feasibility and profitability of IMTA, especially at commercial scale, depends on informed decisions of IMTA producers towards holistic management practices and benthic waste management.
